# Deep Learning-Based Gaze Detection System for Automobile Drivers Using a NIR Camera Sensor

**DOI:** 10.3390/s18020456

**Published:** 2018-02-03

**Authors:** Rizwan Ali Naqvi, Muhammad Arsalan, Ganbayar Batchuluun, Hyo Sik Yoon, Kang Ryoung Park

**Affiliations:** Division of Electronics and Electrical Engineering, Dongguk University, 30 Pildong-ro, 1-gil, Jung-gu, Seoul 100-715, Korea; rizwanali@dongguk.edu (R.A.N.); arsal@dongguk.edu (M.A.); ganabata87@gmail.com (G.B.); yoonhs@dongguk.edu (H.S.Y.)

**Keywords:** eye gaze tracking, driver attention, NIR camera sensor, deep learning, user calibration

## Abstract

A paradigm shift is required to prevent the increasing automobile accident deaths that are mostly due to the inattentive behavior of drivers. Knowledge of gaze region can provide valuable information regarding a driver’s point of attention. Accurate and inexpensive gaze classification systems in cars can improve safe driving. However, monitoring real-time driving behaviors and conditions presents some challenges: dizziness due to long drives, extreme lighting variations, glasses reflections, and occlusions. Past studies on gaze detection in cars have been chiefly based on head movements. The margin of error in gaze detection increases when drivers gaze at objects by moving their eyes without moving their heads. To solve this problem, a pupil center corneal reflection (PCCR)-based method has been considered. However, the error of accurately detecting the pupil center and corneal reflection center is increased in a car environment due to various environment light changes, reflections on glasses surface, and motion and optical blurring of captured eye image. In addition, existing PCCR-based methods require initial user calibration, which is difficult to perform in a car environment. To address this issue, we propose a deep learning-based gaze detection method using a near-infrared (NIR) camera sensor considering driver head and eye movement that does not require any initial user calibration. The proposed system is evaluated on our self-constructed database as well as on open Columbia gaze dataset (CAVE-DB). The proposed method demonstrated greater accuracy than the previous gaze classification methods.

## 1. Introduction

Traffic accidents are mainly caused by a diminished driver vigilance level and gaze distraction from the road [1,2]. Driver distraction is the main source of attention divergence from the roadway and can pose serious dangers to the lives of drivers, passengers, and pedestrians. According to the United States Department of Transportation, 3179 people were killed and 431,000 injured in 2014 due to distracted drivers [3]. Any activity that can divert driver attention from the primary task of driving can lead to distracted driving. It can happen for many reasons, but the most common are using a smart phone, controlling the radio, eating and drinking, and operating a global positing system (GPS). According to the National Highway Traffic Safety Administration (NHTSA) the risk factor for auto wrecks increases three times when drivers are using their smart phones during driving [4]. Using a smart phone causes the longest period of drivers taking their eyes off the road (EOR). In short, it can be a reason for driver distraction, and the technology of driver gaze detection can play a pivotal role in helping to avoid auto accidents. The classification of driver gaze attention is an area of increasing relevance in the pursuit of accident reduction.

Current road safety measures are approaching a level of maturity with the passage of time. One of the major contributions to this is the development of advanced driver assistance systems (ADAS) that can monitor driver attention and send alerts to improve road safety and avoid unsafe driving. Real-time estimation of driver gaze could be coupled with an alerting system to enhance the effectiveness of the ADAS [5]. However, these real-time systems are faced with many challenges for obtaining reliable EOR estimation and classification of the gaze zones. Some significant challenges include: varying illumination conditions; considerable variation in pupil and corneal reflection (CR) due to driver head position and eye movements; variations in physical features that may differ due to gender, skin color, ethnicity, and age; providing consistent accuracy for people wearing glasses or contact lenses; and designing a system for a calibration-free environment. Some of the previous studies have proven to be good under specific conditions, but they have limitations in actual car environments.

To overcome the limitations of previous systems and address the above-mentioned challenges, we propose a near-infrared (NIR) camera sensor-based gaze classification system for car environments using a convolutional neural network (CNN). It is an important issue as this research area has many applications. The proposed system can be used for reliable EOR estimation and ADAS. It uses state-of-the-art deep-learning techniques to solve gaze tracking in an unconstrained environment. 

The remainder of this paper is organized as follows. In Section 2, we discuss in detail the previous studies on gaze detection. In Section 3, the contributions of our research are explained. Our proposed method and its working methodology overview are explained in Section 4. The experimental setup is explained in Section 5, and the results are presented. Section 6 shows both our conclusions and discussions on some ideas for future work.

## 2. Related Works

Several studies have been conducted relating to the gaze classification systems [6,7,8,9]. Gaze classification can be broadly categorized into indoor desktop environments and outdoor vehicle environments. The former can be further divided into wearable device-based methods and non-wearable device-based methods. Wearable device-based methods include a camera and illuminator mounted on the subject’s head in the form of a helmet or a pair of glasses [10,11,12,13,14,15]. In [13,14], a mouse and a wheel chair are controlled by a head-mounted wearable eye-tracking system. Galante et al. proposed a gaze-based interaction system for patients with cerebral palsy to use communication boards on a 2D display. They proposed a system using a head-mounted device with two cameras for eye tracking and frontal viewing [15]. In wearable systems, the problem of absolute head position can be easily avoided as wearable devices move along with head movements. However, the problem of user inconvenience arises when wearing the devices for long periods of time. To address this issue, the non-wearable device-based methods use non-wearable gaze-tracking devices such as cameras and illuminators to acquire face or eye images for gaze tracking [16,17,18,19,20]. Su et al. proposed a gaze-tracking system that was based on a visible light web camera. In this system that detected a face on the basis of skin color, luminance, chrominance, and edges, eyes are tracked to control the mouse [16]. A gaze-tracking system for controlling such applications as spelling programs or games was proposed by Magee et al. [17] A remote gaze detection method was proposed by Lee et al. that uses wide- and narrow-view cameras as an interface for smart TVs [18]. In addition, a typical example of the non-wearable eye-tracking method is the PCCR-based method [19,20]. One major advantage of PCCR-based methods is they require no complicated geometrical knowledge about lighting, monitors, cameras, or eyes. User convenience of non-wearable device-based methods is higher than that of the wearable gaze-tracking methods, but initial user calibration or camera calibration is required to map the camera, monitor, and user’s eye coordinates. In addition, these studies have focused only on indoor desktop environments considering small-sized monitors. In this study, we try to analyze the applicability of PCCR-based methods in a vehicle environment in which the head rotation of user is larger than that in desktop monitor environments and initial user calibration is difficult to perform.

The second category includes outdoor vehicle environments to classify the driver’s gaze position and their behavior while driving. Rough gaze position based on driver head orientation is usually acceptable in driver behavior analyzing systems. Gaze zone estimators are being used to generate the probability of driver attention position. Outdoor vehicle environments for gaze classification can be further divided into two categories: multiple camera-based methods and single camera-based methods.

In past research, multiple camera-based methods were mostly used for the outdoor vehicle environment [21,22,23,24]. When dealing with the challenges of more peripheral gaze directions or large gaze coverage, multiple cameras may be the most suitable solution. Ahlstrom et al. [21] used multi-camera eye trackers in the car environment. They installed two hidden cameras, one at the A-pillar and one behind the center console of the car for covering forward-facing eye gazes. They tried to investigate the usefulness of a real-time distraction detection algorithm named AttenD. The performance, reliability, and accuracy of AttenD is directly dependent on eye-tracking quality. In addition, they defined the field relevant for driving (FRD) excluding the right-side mirror, but it is often the case that drivers gaze at the right-side mirror while driving.

Liang et al. observed driver distraction by using eye motion data in a support vector machine (SVM) model [22]. They compared it with a logistic regression model and found the SVM model performed better in identifying distraction. However, wearing glasses or eye make-up can adversely affect the accuracy of this system. An initial calibration of 5 to 15 min is also required and can be time-consuming and annoying for drivers. The concept of a distributed camera framework for gaze estimation was given by Tawari et al. [23]. They tracked facial landmarks and performed their correspondence matching in 3D face images. A random forest classifier in combination with proposed feature set was used for zone estimation. Since a visible light camera is used instead of a NIR light camera, it is greatly influenced by the external light conditions. Although the accuracy of the driver’s gaze was high, only eight frontal gaze regions were considered. Later they proposed [24] that head pose for gaze classification can be estimated effectively by facial landmarks and their 3D correspondences. This is done by using a pose from an orthography and scaling (POS) algorithm [25]. Later, they used a constrained local model (CLM) to extract and analyze the head pose and its dynamic in the multi-camera system [26]. Although multiple camera-based methods show high accuracies of gaze estimation, the processing time is increased by the images of multiple cameras.

Considering this issue, single camera-based methods have been researched [27,28,29,30,31]. SVM was used by Lee et al. to estimate driver gaze zones by using their pitch and yaw clues [27]. The camera resolution of this system [27] is low with low illuminator power, and the driver’s pupil center cannot be detected. Therefore, they estimated the driver’s gaze position only by measuring head rotation (not eye rotation), and obtained the experimental data by instructing drivers to rotate their heads intentionally and sufficiently. If a driver only moves the eyes to gaze at some position without head rotation (which is often the case while driving), their method cannot detect driver gaze position. Vicente et al. [28] proposed a supervised descent method (SDM) using a scale invariant feature transform (SIFT) descriptor to express face shape by providing a clear representation against illumination. For eye pose estimation, facial feature landmarks use eye alignment to locate the eye region. There is an advantage that the camera position is not significantly affected by the change. However, the disadvantage is that the driver’s wide head rotation and the use of thick glasses can decrease its performance. In addition, accuracy of the gaze tracker system may be limited as pupil center position in the driver’s image can be mixed with the iris center position in daylight when not using the NIR light illuminator [32]. In [29,30], they detected skin regions based on pre-trained skin color predicate by Kjeldsen et al.’s method [33]. In the detected image, the eye region is classified as the non-skin region. If a non-skin region is detected above the lip corners, it becomes the most probable eye region. A small window is set and searched within the determined eye region to determine the pupil with the lowest pixel value, and the eye is traced using the optical flow algorithm of [34]. Finally, assuming that the eyes are aligned, they estimated the driver’s gaze by modeling head movements using the position of both eyes, the back of the head just behind both eyes, and the center of the back of the head. In this study, it is not necessary to measure the distance from the driver’s head to the camera, but this is because it detects only the direction of the eyes. When the driver’s head and eyes rotate in opposite directions, or when the head does not rotate and only the eyes move, accuracy of tracking is decreased. In addition, since the iris center position is detected instead of the pupil center position, there is a limitation in improving the accuracy of eye tracking. Fridman et al. combined the histogram of oriented gradients (HOG) with a linear SVM classifier to find face region and classify feature vectors to gaze zones by random forest classifier [31]. Previous studies use Purkinje images [35] or detect facial feature points [36] to estimate gaze. Purkinje images (PI) are the light reflections generated on the cornea and crystalline lens of the human eye [37]. By analyzing the movements of these reflections (especially, the 1st and 4th PI), it is possible to identify the direction of eye rotation and determine gaze. However, this study did not evaluate the gaze detection accuracy in a vehicular environment [35]. Fridman, et al. [36] used facial feature points to find the iris and binarized it to estimate the area being gazed at. However, their accuracy was not high, because the iris could be detected in only 61.6% of the test images. There were limits to enhancing the accuracy of gaze detection because the center of the iris, and not the pupil, was detected. Choi et al. [38] detected driver faces with a Haar feature face detector and used CNN to categorize gaze zones, but they considered only eight gaze regions. Vora et al. proposed the method of driver’s gaze estimation by CNN, but small numbers of gaze regions (six gaze regions) were considered in this research [39]. Therefore, the detailed gaze position of the driver cannot be detected. Fu et al. proposed automatic calibration method for the driver’s head orientation by a single camera [40]. However, their calibration method requires the driver to gaze at several positions such as the side mirrors, the rear-view mirror, the instrument board, and different zones in the windshield as calibration points, which causes inconvenience to the driver in the actual car environment. In addition, only 12 gaze zones were considered in their research.

Other categories of gaze detection methods, such as regression-based methods, have been studied including appearance-based gaze estimation via uncalibrated gaze pattern recovery and adaptive linear regression for appearance-based gaze estimation [41,42]. 

Ghosh et al. [43] proposed using eye detection and tracking to monitor driver vigilance. However, their method classified open or closed eyes instead of detecting the driver’s gaze position. In addition, the camera angle is small, and there is the limitation of movement of the driver’s head, which causes inconvenience to the driver. García et al. [44] proposed a non-intrusive approach for drowsiness detection. Cyganek et al. proposed the hybrid visual system for monitoring the driver’s states of fatigue, sleepiness and inattention based on the driver’s eye recognition using the custom setup of visible light and NIR cameras and cascade of two classifiers [45]. Chen et al. proposed the method of detection of alertness and drowsiness by fusing electroencephalogram (EEG) and eyelid movement by electrooculography (EOG) [46]. However, as with the research in [43], their method [44,45,46] just recognized the alertness/drowsiness status of the driver by classifying open or closed eyes (or by physiological signals), not by detecting the driver’s gaze position. Kaddouhi et al. proposed the method of eye detection based on the Viola and Jones method, corner points, Shi-Tomasi detector, K-means, and eye template matching [47]. This is just for the research of eye detection, and driver’s gaze position was not detected in this research.

In previous research [48,49,50,51], they investigated the drivers’ visual strategies, the distribution of fixation points, driving performance, and gaze behavior by on-road experiment or driving simulator. Their research was focused on the analyses of the driver’s visual characteristics while driving instead of proposing new gaze detection methods.

Considering the limitations of existing studies, we investigated a method for driver gaze classification in the car environment using deep CNN. In Table 1, we have summarized the comparison of the proposed method and existing methods on gaze classification in vehicle environment.

## 3. The Contributions of Our Research

Our research has contributed in the following four ways compared to previous works.

- Although there exists only one previous piece of research that used a shallow CNN of AlexNet for gaze estimation in the car environment [38], they used only one CNN using whole face image as input for estimating the small number of gaze regions (8 regions). Because the accurate gaze position of the driver cannot be detected based on only these 8 regions, we increased the number of gaze regions into 17 as shown in Figure 3a. The consequent classification complexity of gaze estimator is increased, and one shallow CNN using one input face image cannot show good accuracy of gaze estimation as shown in Tables 10 and 13. Therefore, we use three deep CNNs that use the images of left eye, right eye and face, respectively, and combine the outputs by these three CNNs based on score level fusion, which shows higher accuracy of gaze estimation without initial driver calibration.
- In order to check the effect of PCCR vectors on the accuracy of gaze estimation, the performance by our method based on three CNNs, which use the images of left eye, right eye and face is compared to the three CNNs with PCCR vectors.
- Through the fine tuning of a pre-trained visual geometry group (VGG) CNN model, accurate gaze detection can be done without intensive training (scratch training) of the whole CNN model, which can reduce the number of training data.
- We make our collected database and trained CNN model open to other researchers through [52] to enable them to have comparisons with our database and model.

## 4. Gaze Classification in Outdoor Vehicle Environment Using CNN

### 4.1. Overview of Proposed Method

**Figure 1:**
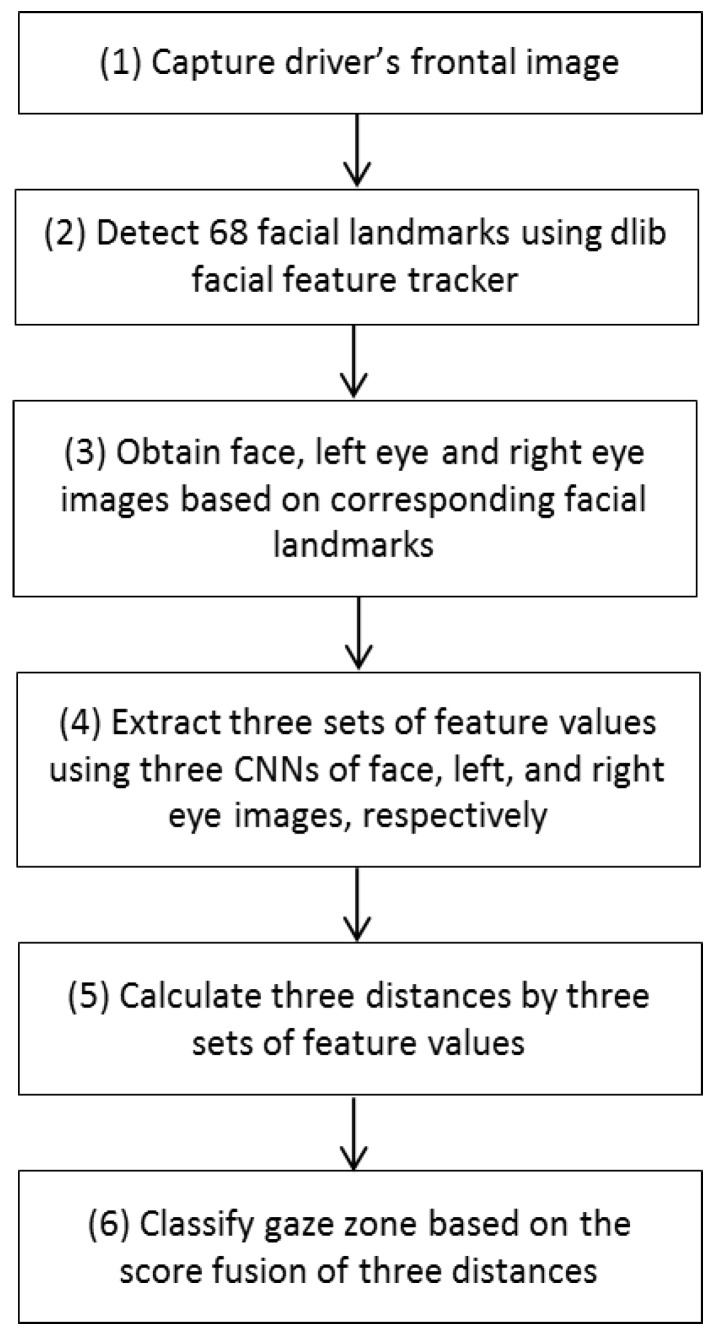
Flowchart of proposed system.

**Figure 2:**
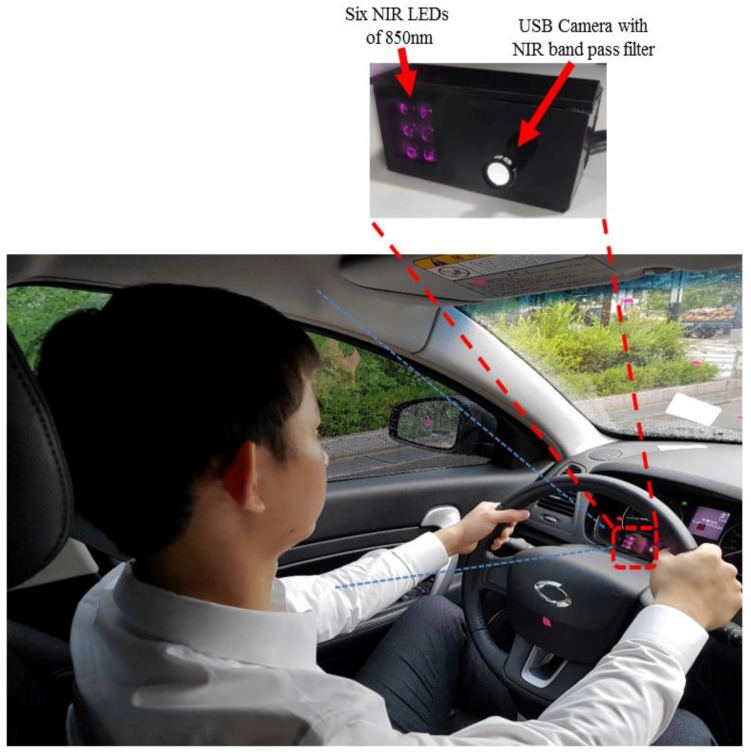
Experimental environment and proposed gaze detection system in vehicle environment.

An overview of the system is shown in Figure 1. After the NIR camera of our system of Figure 2 captures the image frames of the driver’s frontal view, 68 face landmarks are detected by the Dlib facial feature tracker [53] (steps (1) and (2) of Figure 1, and details are explained in Section 4.2). Then, the region-of-interest (ROI) images of face, left and right eye are obtained based on the corresponding face landmarks position (step (3) of Figure 1). Brightness normalization is done in each ROI image based on the mean of all the pixel values in each ROI image to increase the performance of the system and reduce the effect of light. In step (4) of Figure 1, three sets of feature values are extracted using three CNNs of face, left, and right eye ROI images, respectively (details are explained in Section 4.4). Then, each set of feature values is normalized, and three distances are calculated by three sets of feature values (step (5) of Figure 1). Here, distance is calculated between the input set of feature values and that in each gaze zone. Finally, our system classifies the driver’s gaze zone based on score fusion of three distances (details are explained in Section 4.4.4).

**Figure 3:**
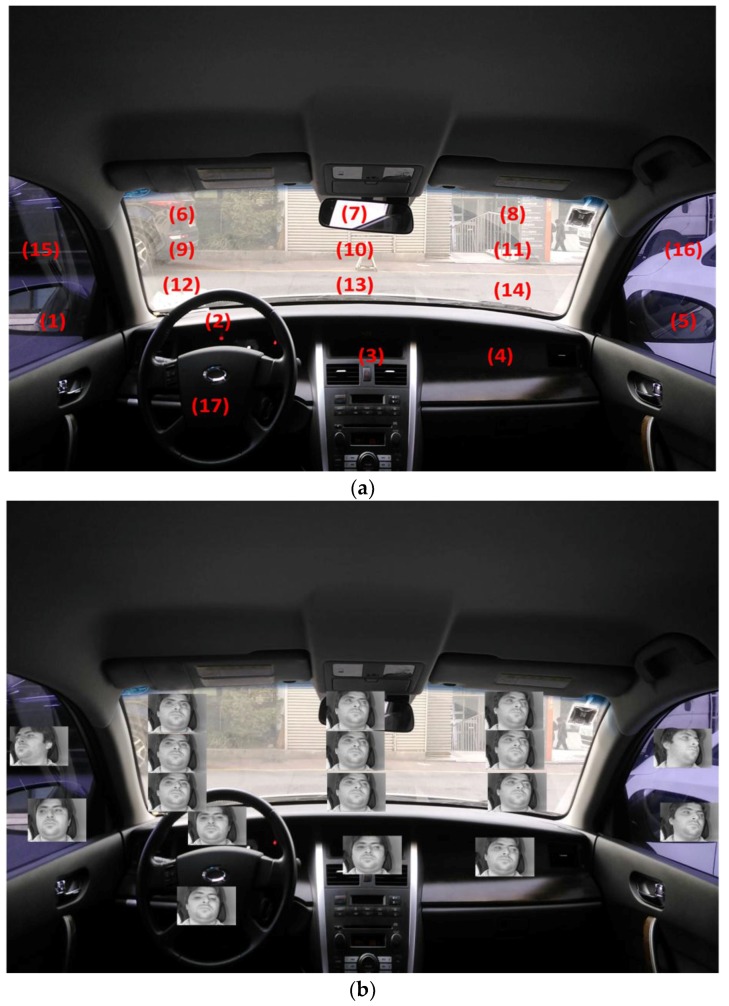
17 gaze zones in our experiments. (**a**) 17 gaze zones; (**b**) Captured images when the driver looked at each gaze zone.

Figure 2 and Figure 3 show our experimental environment with proposed device of gaze detection and 17 gaze zones in our experiment, respectively. As shown in Figure 2, our device consists of a NIR camera and the illuminator of 6 NIR light emitting diodes (LEDs). Because the size of the device is small (8.8 cm × 4.3 cm × 4.0 cm), it could be installed in the vicinity of the dashboard, as shown in Figure 2, and can continuously track the driver’s gaze without obscuring the dashboard. The NIR illuminator in the gaze detection system was placed to the left of the camera, and helped capture the driver’s facial image without being influenced by changing ambient light. Using the NIR LEDs at a wavelength of 850 nm, which is a little visible to the driver’s eye, prevented uncomfortable situations, such as the driver being blinded or fatigued by the light while driving, and it distinguished the boundary of the pupil. A zoom lens (focal length of 9 mm) was attached to the camera to capture enlarged facial images of the driver. An 850-nm band pass filter (BPF) was also mounted on the camera’s lens to minimize interference due to sunlight [54]. Power to this device was supplied by a laptop computer using two universal serial bus (USB) lines, one connecting the camera and the other the illuminator. The captured image by web camera of our gaze-tracking device is successively transmitted to the laptop computer via a USB interface line. The characteristics of the camera and the illuminator of our gaze detection system are shown in Table 2.

Data were obtained and processed on a laptop computer with 2.80 GHz CPU (Intel^®^ Core ™, Santa Clara, CA, USA, i5-4200H) and 8 GB of RAM.

### 4.2. Detecting Facial Landmarks by Dlib Facial Feature Tracker

**Figure 4:**
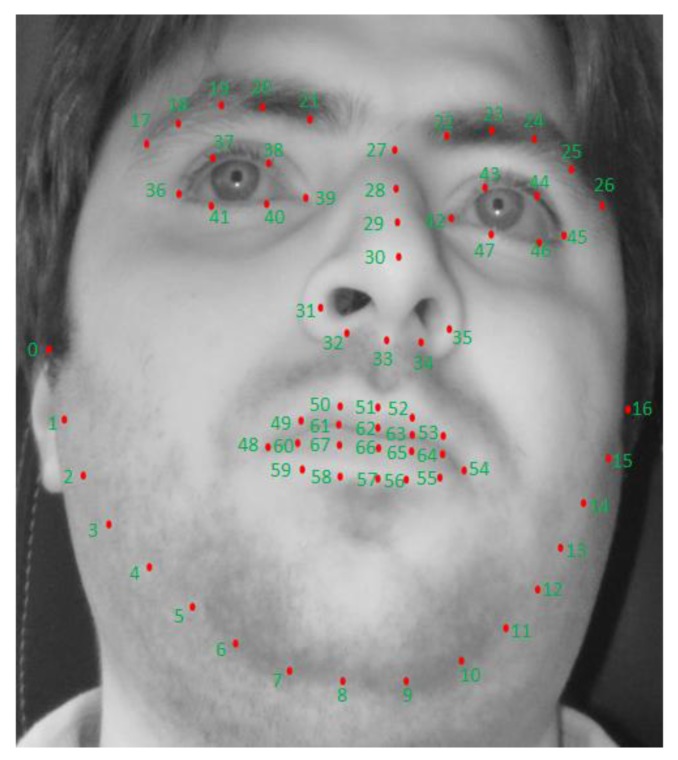
Example of detected facial landmarks.

In our research, 68 facial landmarks are detected by the Dlib facial feature tracker [53]. Facial landmarks are used to localize and represent salient regions of the face, such as eyes, eyebrows, nose, mouth and jawline. It can be successfully applied to various applications of face alignment, face swapping, and blink detection etc. In our case we have utilized it to extract face, left eye, and right eye areas. The procedure of obtaining facial landmarks is composed of localizing the face in the image and detecting the main facial structures on the face ROI. Localizing the face in the image can be done various ways such as using Haar cascades detector, HOG and Linear SVM-based detector, and deep learning-based algorithms. In any case, the major purpose is to find the face-bounding box. Once the face is localized through face-bounding box, our next target is to detect key facial structures in the face area. The Dlib facial feature tracker is used to estimate 68 (x,y)-coordinates that are mapped on the facial structure of the face. The indices of the 68 coordinates of facial landmarks are shown in Figure 4.

### 4.3. Calculating PCCR Vector for Left and Right Eye

For showing the effectiveness of our proposed method, we have made comparison of a method considering PCCR vector (scheme 2) with our proposed method without considering PCCR (scheme 1). For this purpose, we calculated PCCR vector from left and right eyes. There have been various previous studies on eye, pupil, and CR detection [56,57]. In our research, within the captured eye ROI, defined based on the facial landmarks of 36~41 (for left eye) and those of 42~47 (for right eye) of Figure 4, the pupil center and CR center are detected as follows [7]. As the first step, histogram stretching is performed within the eye ROI. Then, the image subjected to histogram stretching goes through image binarization. This is intended to distinguish the pupil from the non-pupil regions as well as the CR from the non-CR regions. Morphological processing and component labeling are used on the binarized image to find the largest region. Then, based on the boundary identified using the canny edge detector, the outer boundary of the pupil is detected in the image by using the convex hull algorithm. By subtracting the overlapping area (of this boundary and the binarized CR region) from the outer boundary, it is possible to find the pupil boundary that is not distorted by the CR. Finally, the pupil center is accurately detected by performing ellipse fitting based on this boundary. 

**Figure 5:**
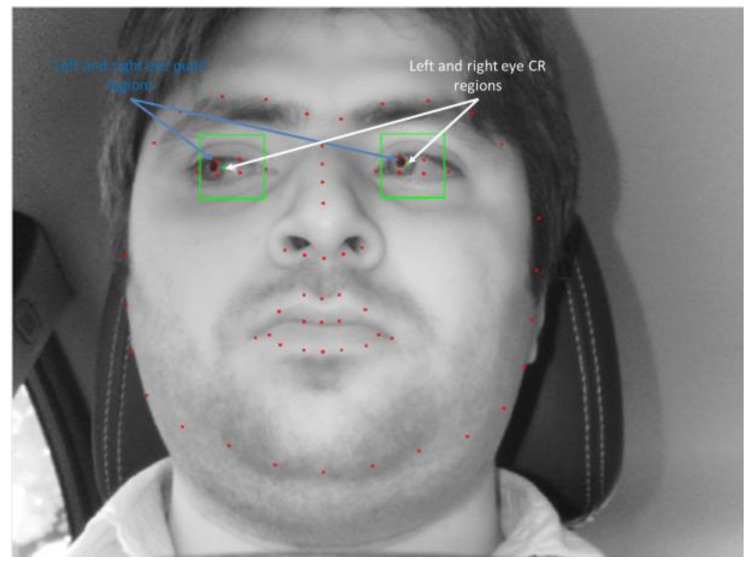
Example of detected pupil and CR regions.

A search region is defined to detect the CR centered on the detected center of the pupil. Image binarization is performed on this search region to distinguish the CR and non-CR regions, after which component labeling is performed on the non-CR region because areas with the same pixel brightness as the CR can still exist in the region. The region closest to the detected pupil center is then designated as the CR region, and the geometric center of the designated CR is determined to be the CR center. An example of the detected pupil and CR regions are shown in Figure 5.

**Figure 6:**
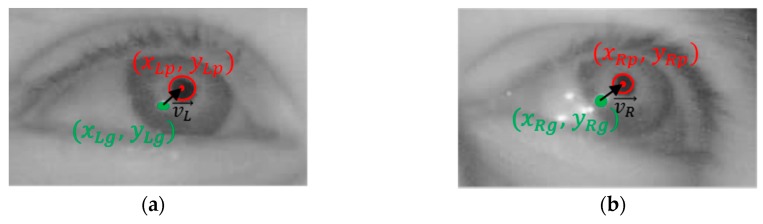
The PCCR vectors generated from (**a**) left eye and (**b**) right eye images.

Based on the detected two centers of the pupil and CR, the PCCR vector is calculated. The PCCR vector is most commonly used to calculate gaze position [7,56]. Figure 6 shows a typical pupil–corneal reflection setup. Visual axis angle is calculated by tracking the relative position of the pupil center and CR technically known as “glint”. Assuming that the positions of camera and light source are fixed, the eye is a sphere that only rotates around its center, and the position of CR does not move with the eye rotation, CR (glint) can be suitable as a reference point. Therefore, 2D PCCR vectors are calculated as $\vec{v_{L}}$ and $\vec{v_{L}}$, respectively, as shown in Equation (1) and Figure 6.

(1) $$\vec{v_{L}}=x_{Lp}-x_{Lg},\;y_{Lp}-y_{Lg}\;\vec{v_{R}}=x_{Rp}-x_{Rg},\;y_{Rp}-y_{Rg}$$

where ($x_{Lp},\;\;y_{Lp}$) and ($x_{Rp},\;\;y_{Rp}$) are the two pupil centers of left and right eye image, respectively, whereas ($x_{Lg},\;\;y_{Lp}$) and ($x_{Rg},\;\;y_{Rg}$) are the two CR (glint) centers of left and right eye image, respectively.

### 4.4. Driver’s Gaze Classification Based on CNN

#### 4.4.1. Gaze Feature Extraction

**Figure 7:**
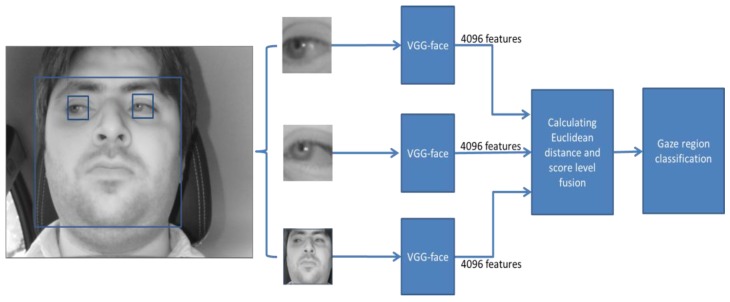
CNN-based driver’s gaze classification procedure without PCCR vector (scheme 1).

For extracting gaze feature, we use three inputs extracted from the driver’s image, the face, left eye, and right eye. For obtaining these inputs, we used the detected 68 landmarks on the face of Figure 4. We added a margin of 50 pixels around the outer landmarks of face, left eye, and right eye to crop face, left eye, and right eye ROI images around them. Once the input images are cropped they are resized to the images of 224 × 224 pixels using bi-linear interpolation, and they are used as inputs to three CNNs as shown in Figure 7 (scheme 1).

In this research, we used the original VGG-face network (including 16 layers) that was trained for face recognition [58]. The VGG-face 16 model was trained using approximately 2.6 million face images obtained from 2622 people. The structure of the VGG-face 16 model is similar to the VGG-Net 16 architecture [59], and their accuracies were evaluated on the databases of Labeled Faces in the Wild (LFW) [60] and YouTube Faces (YTF) [61]. With this model of VGG-face 16, we performed the fine-tuning with our training data. Detailed explanations on the training and testing data are shown in Section 5.1. With the fine-tuned VGG-face model, we extracted features from the response of the fully connected layer (FCL), which is the second to last level (Fc7 of Table 3) of 4096-dimensional descriptor. After extracting three sets of features of 4096 from face, left eye, and right eye images, the final gaze zone among 17 zones of Figure 3a is determined based on the minimum distance, and detailed explanations are included in Section 4.4.4.

**Figure 8:**
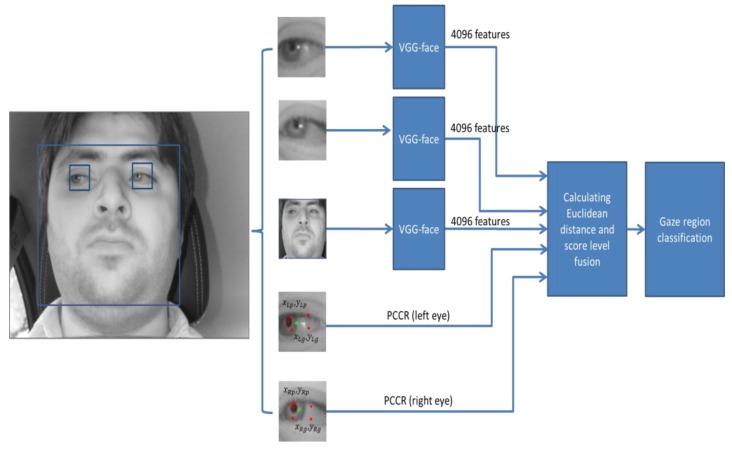
CNN-based driver’s gaze classification procedure with PCCR vector (scheme 2).

As the next scheme (scheme 2 of Figure 8), we also considered the PCCR vector for gaze zone classification because the PCCR vector has been widely used for previous studies on gaze detection. Then, we compared the performance of scheme 1 of Figure 7 with the scheme 2 of Figure 8 for calculating the driver’s gaze classification system. Like scheme 2, after extracting three sets of features of 4096 from face, left eye, and right eye images with two additional PCCR vectors from left and right eye, respectively, final gaze zone among 17 zones of Figure 3a was determined based on the minimum distance, and detail explanations are also included in Section 4.4.4.

#### 4.4.2. CNN (VGG-Face 16) Structure

**Figure 9:**
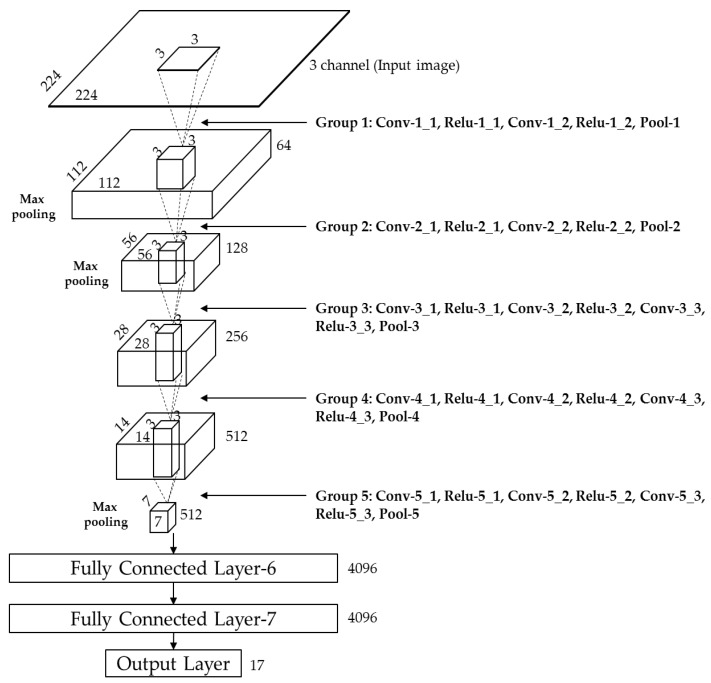
CNN architecture used for finding required gaze features.

We are going to discuss our CNN structure that is represented in Figure 9 and explained in Table 3. It is comprised of 13 convolutional layers, 5 pooling layers, and 3 fully connected layers (FCLs). In the first convolutional layer, 64 filters of the size of 3 × 3 are used for the input of 224 × 224 × 3. Here, 224 × 224 × 3 represents width, height, and number of channel, respectively. From that, the feature map of 224 × 224 × 64 is obtained. This can be calculated based on the following criteria: (output width (or height) = (input width (or height) − filter width (or height) + 2 × the padding number)/the stride number + 1) [62]. For example, in Table 3, the input width, filter width, the padding number, and the stride number are 224, 3, 1, and 1 respectively. Therefore, the output width of the feature map by convolution is calculated as 224 (= (224 − 3 + 2 × 1)/1 + 1). In general, the output feature map for standard convolution based on stride one and padding is obtained by [63]:

O_*k,l,n*_ = Σ_*i,j,m*_ K_*i,j,m,n*_ ⋅ I_*k+i*−1,*l+j*−1,*m*_ (2)

When I*_k+i_*_−1*,l+j*−1*,m*_ is the input feature map of the size of *S_F_* × *S_F_* × *P*. *S_F_* is the width and height of square input feature map, and *P* is the number of input channels (input depth). O*_k,l,n_* is the output feature map of the size of *T_F_* × *T_F_* × *Q*. *T_F_* is the spatial width and height of a square output feature map, and *Q* is the number of output channels (output depth). K*_i,j,m,n_* is the convolution kernel of size *S_K_* × *S_K_* × *P* × *Q*, and *S_K_* is the spatial dimension of convolution kernel. From that, standard convolutions have the following computational cost of:

*C* = *S_K_* · *S_K_* · *P* · *Q* · *S_F_* · *S_F_* (3)

Based on Equation (2), we can find that the computational cost depends multiplicatively on the kernel size *S_K_* × *S_K_*, the number of input channels *P*, the number of output channels *Q*, and the input feature map size *S_F_* × *S_F_* [63].

Rectified linear unit (ReLU) layer is based on the following function as shown in Equation (4) [64,65].

(4) y=max(0,x)

In Equation (4), x and y are the input and output values, respectively. This function reduces the problem of vanishing gradient [66] that may occur when a hyperbolic or sigmoid tangent function is used in back-propagation for training. In addition, it has a faster processing speed than a non-linear activation function. After passing through the ReLU layer (ReLU-1_1 of Table 3), the feature map obtained through the second convolutional layer is once again passed through the ReLU layer (ReLU-1_2 of Table 3) before passing through the max pooling layer (Pool-1 of Table 3) as shown in Table 3. Here, the 2nd convolutional layer maintains the feature map size of 224 × 224 × 64 with filter of size 3 × 3, padding 1 × 1, and stride 1 × 1 as in the first convolutional layer.

The maximum value among the values defined in the filter range is selected in the max pooling layer, which performs a kind of subsampling. Note that after ReLU-1_2 of Table 3, the feature map size is 224 × 224 × 64. By using max pooling layer (Pool-1) of kernel size of 2 × 2, and stride of 2 × 2, the feature map size is reduced to 1/4 (112 × 112 × 64) because there is no overlapping area for the filter. 

As shown in Table 3, the size of kernel of 3 × 3, the padding number of 1 × 1, and the stride number of 1 × 1 are used in all 13 convolutional layers. Only the number of filters is changed to 64, 128, 256, and 512. Each ReLU layer is followed by a convolutional layer. Similarly, each max pooling layer is used after ReLU-1_2, ReLU-2_2, ReLU-3_3, ReLU-4_3, and ReLU-5_3 in Table 3, and the filter in each max pooling layer is composed of size of 2 × 2, the stride of 2 × 2, and the padding of 0 × 0. As explained, the feature map size is reduced at each max pooling layer, ReLU-1_2 (224 × 224 × 64) is reduced to Pool-1 (112 × 112 × 64), ReLU-2_2 (112 × 112 × 128) to Pool-2 (56 × 56 × 128), ReLU-3_3 (56 × 56 × 256) to Pool-3 (28 × 28 × 246), ReLU-4_3 (28 × 28 × 512) to Pool-4 (14 × 14 × 512), and ReLU-5_3 (14 × 14 × 512) to Pool-5 (7 × 7 × 512). 

#### 4.4.3. FCLs of CNN

Once the input image is passed through the 13 convolutional layers, 13 ReLU layers, and 5 pooling layers, we can get the feature map size of 7 × 7 × 512 pixels. The obtained feature map passes through the additional three FCLs. After each FCL, the feature maps of 4096 × 1, 4096 × 1, and 17 × 1 are obtained, respectively, as shown in Table 3. In this study, we have designed the classification system for driver’s gaze region by CNN. Because the number of gazing zones is 17 as shown in Figure 3a, the output layer of Table 3 is 17 × 1.

In the third FCL, the softmax function is used as shown in Equation (5) [67].

(5) $$\sigma \left(r\right)=\frac{e^{ri}}{\sum_{n=1}^{K}e^{rn}}$$

Here, *r* is an array of output neurons; we can obtain the probability of neurons belonging to the *i*th class by dividing the value of the *i*th element by the summation of all the elements. 

It has been frequently observed that there is the problem of low recognition accuracy with testing data in CNN-based cognitive systems due to over-fitting in the data. To solve this issue, we used data augmentation and dropout methods [68,69]. A detailed description of the experimental data generated by the data augmentation is given in Section 5.1. For the dropout method, we adopt the dropout probability of 50% to randomly disconnect the links between the previous layer and the current layer in the 1st and 2nd FCLs. 

#### 4.4.4. Classifying Gaze Zones by Score Fusion of Three Distances

As explained in Section 4.4.1, after extracting three separate feature sets (three sets of 4096 features) from face, left eye, and right eye images (scheme 1), we normalized them to each other by min-max scaling. With the training data, we already saved the three (normalized) feature sets per each gaze zone of Figure 3a. Then, we can calculate three Euclidean distances between the three feature sets of inputs and the three saved on each gaze zone. After that, these three distances were combined based on score level fusion. Finally, one final score (distance) is obtained, and the gaze zone whose final score (distance) is smallest among 17 zones of Figure 3a is determined as the driver gazing region. As explained in Section 4.4.1, in scheme 2, five feature sets from face, left eye, and right eye images with two additional PCCR vectors from left and right eye are used instead of three feature sets from face, left eye, and right eye images. 

For the score level fusion, the performances of weighted SUM and weighted PRODUCT rules of Equations (6) and (7) were compared, and optimal weights were selected with training data. Detailed explanations are shown in Section 5.3.1.

(6) $$WS=\overset{m}{\underset{i=1}{\sum }}w_{i}d_{i}$$

(7) $$WP=\overset{m}{\underset{i=1}{\prod }}d_{i}^{w_{i}}$$

where *m* is 3 in case of scheme 1; whereas *m* is 5 in case of scheme 2 as shown in Figure 7 and Figure 8. In scheme 1, *i* of 1, 2 and 3 shows the left eye, right eye, and face, respectively, as shown in Figure 7. In scheme 2, *i* of 1~5 represents the left eye, right eye, face, PCCR vector of left eye, and PCCR vector of right eye, respectively, as shown in Figure 7. *WS* and *WP* are respectively the scores by weighted SUM and weighted PRODUCT rules, *d_i_* is the Euclidean distance obtained from the input, and *w_i_* is the weight. 

The 17 outputs of the output layer in Figure 9 represent the 17 gaze regions of Figure 3a. If we use one CNN for gaze estimation, these 17 outputs can be used for the detection of final gaze position. However, in our research, we combine the gaze estimation results by three CNNs (scheme 1 of Figure 7) or three CNNs with two PCCR vectors (scheme 2 of Figure 8). In these cases, the 17 outputs from one CNN cannot be combined with other outputs or PCCR vectors. Therefore, we use 4096 features from Fc7 of Table 3 for obtaining the combined Euclidean distance by score level fusion, by which the final gaze position can be detected as shown in Figure 7 and Figure 8.

## 5. Experimental Results

### 5.1. Experimental Data and Environment

**Figure 10:**
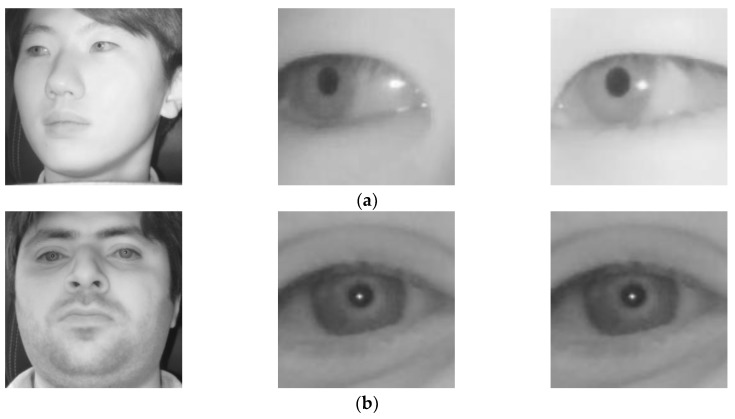

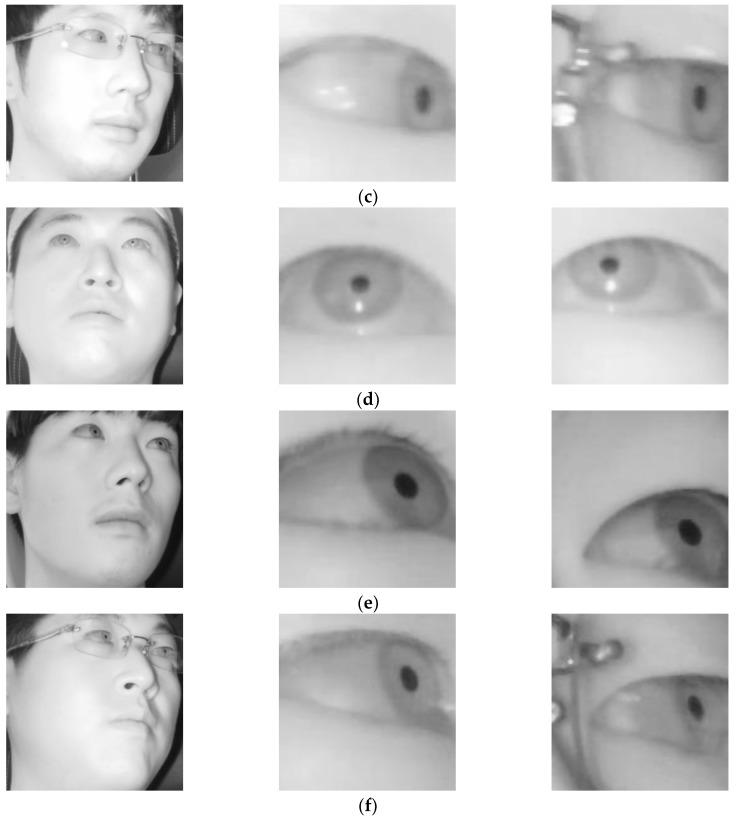
Example images of face (**left**), left eye (**middle**), and right eye (**right**) while looking at different regions of Figure 3. Cases of looking at (**a**) region 1; (**b**) region 2; (**c**) region 5; (**d**) region 6; (**e**) region 7; and (**f**) region 8.

In this research, we have collected our own database (DDGC-DB1) for the driver’s gaze classification system in the car environment as shown in Figure 10. It was obtained through the experimental setup that can be viewed in Figure 2. Most previous driving databases are not open access for academic research as they are prepared by auto manufacturers. The Chinese Academy of Sciences pose, expression, accessories, and lighting (CAS-PEAL) database is very popular and widely used for baseline evaluation of gaze estimation or face recognition with various factors of pose and light [70]. However, this database was collected in a laboratory instead of an actual car environment, and various factors in cars are not reflected in this database. Another database is RobeSafe driver monitoring video (RS-DMV) dataset [71]. However, this database does not fit to our purpose because the information of ground-truth gaze position is not provided. Without this information, we cannot evaluate the accuracies of our gaze detection method. Therefore, we collected our own database (DDGC-DB1).

As shown in Figure 3, 17 spots (gaze zones) were designated to gaze at for the experiment, and each driver stared at each spot five times. Data were collected from 20 drivers including 3 wearing glasses. The image size is 1600 × 1200 pixels with 3 channels. When the participants were staring at each spot, they were told to act normally, as if they were actually driving and were not restrained to one position or given any special instructions to act in an unnatural manner. There were risks of car accidents to motivate the participants to accurately stare at the 17 designated spots while actually driving for the experiment. Instead, this study obtained images from various locations (from roads in daylight to a parking garage) in a real vehicle (model name of SM5 New Impression by Renault Samsung [72]) with its power on, but in park to create an environment most similar to when it is being driven (including factors such as car vibration and external light). Moreover, to understand the influence of various kinds of external light on driver gaze detection, test data were acquired at different times of the day: in the morning, the afternoon, and at night. From our database, we obtained the images (of 224 × 224 pixels) of face, left, and right eyes for CNN training and testing as shown in Figure 10.

The research by Lee et al. [27] used 18 gaze zones by using an additional zone (the upper position of region 6 of Figure 3) compared to 17 zones in our research. However, the case of gazing at this additional zone does not frequently occur while driving [48,49,50,51]. Therefore, in previous studies, they did not use this additional zone for experiments, either [24,36,38,39,40]. Based on that, we did not use this additional zone, and performed the experiments with the data where drivers gazed at the 17 positions of Figure 3.

**Figure 11:**
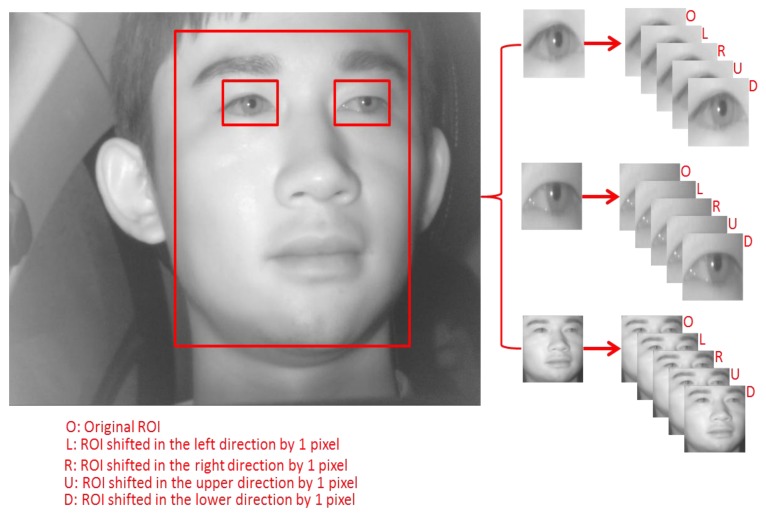
Augmented images obtained from the original ROI image.

In our experiment, we performed two-fold cross validation for training and testing. For that, we have randomly divided our databases into two subsets of face, left eye, and right eye images as shown in Table 4. Then, as explained in Section 4.4.3, data augmentation with training data is performed to avoid the overfitting problem as follows. Five images are obtained from each rectangular ROI defined for the face, left eye, and right eye in the image by shifting 1 pixel in the left, right, up and down directions based on the coordinates of the original image. Hence five images are obtained from each face, left eye, and right eye in the single original image as shown in Figure 11. Original data was used for testing whereas the augmented data was used only for training as shown in Table 4. 

For the CNN training and testing, we used a desktop computer with an Intel^®^ Core™ (Santa Clara, CA, USA) i7-3770K CPU @ 3.50 GHz, 16 GB memory, and a NVIDIA GeForce GTX 1070 (1920 CUDA cores and 8 GB memory) graphics card [73]. Our algorithm was implemented by Microsoft Visual Studio 2013 C++, and OpenCV (version 2.4.5) [74] library and Boost (version 1.55.0) library. The training and testing algorithm of the CNN model was implemented by Windows Caffe (version 1) [75].

### 5.2. Training of CNN Model

Stochastic gradient descent (SGD) method was used for CNN training [76]. The SGD method is a derivative-based method of finding the optimal weight that minimizes the difference between the desired output and the calculated output. Unlike the gradient descent (GD) method, in the SGD method, the number of training sets divided by mini-batch size is defined as iteration, and one epoch is set when training is performed for all the number of iterations as shown in Equations (8) and (9) [69].

(8) $$v_{i+1}:\;=m\;\cdot v_{i}-d\cdot \eta \cdot w_{i}-\eta \cdot \langle \frac{\partial Q_{i}\left(w\right)}{\partial w}|w_{i}\rangle D_{i}$$

(9) $$w_{i+1}:\;=w_{i}+v_{i+1}$$

where $w_{i}$ is the weight to be learnt at the *i*th iteration. *m* is momentum, $v_{i}$ is the momentum variable, *d* is the weight decay, and η is the learning rate. $\langle \frac{\partial Q_{i}\left(w\right)}{\partial w}|w_{i}\rangle D_{i}$ is the average over the *i*th batch *D_i_* of the derivative of the object with respective to w, evaluated at $w_{i}$. In our experiment, the training was performed for the predefined epoch count of 16 based on the maximum number of training i.e., about 13,048. *m*, *d*, and η of Equations 8 and 9 were set as 0.9, 0.0005, and 0.00001, respectively, with batch size of 20.

**Figure 12:**
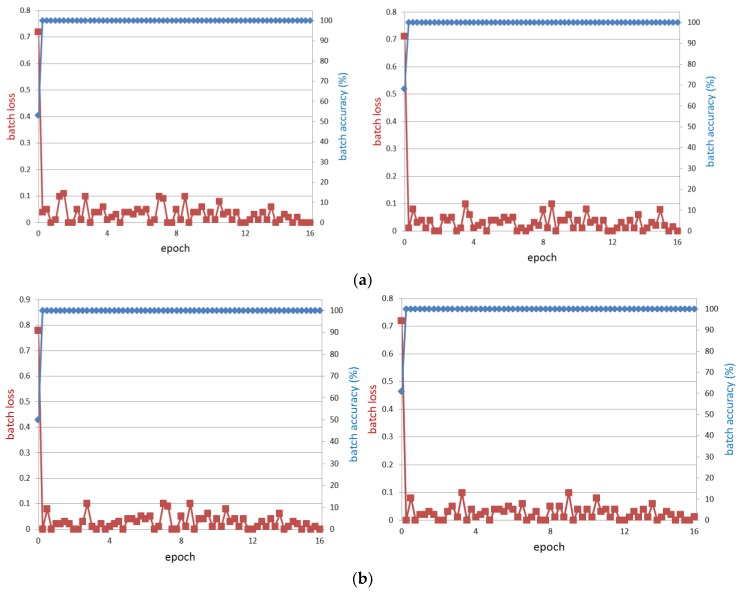

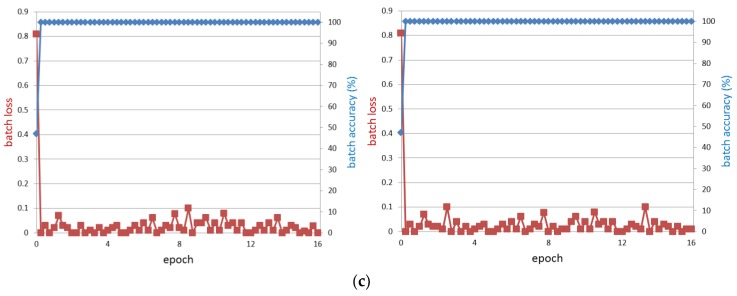
The curves of training loss and training accuracies according to the number of epoch with the sub-databases of (**a**) face; (**b**) left eye; and (**c**) right eye. In (**a**–**c**), left and right figures respectively show the graphs from the training of first- and second-fold cross validations.

Figure 12 shows the visualization of the relationship between loss and training accuracy during training of sub-databases of face, left eye, and right eye. The x-axis represents the number of epoch. The left side of the y-axis represents the loss and the right side of the y-axis represents the training accuracy. The loss depends on the learning rate and batch size. When the learning rate is lowered, it slowly goes down, showing linearity. If the learning rate is high, the loss decreases sharply, but the loss value changes suddenly, which may lead to the problem of maintaining the loss value without reaching the optimal CNN model. In this experiment, we used optimal models with loss curves close to 0 (0%) and training accuracies close to 1 (100%) as shown in Figure 12. 

### 5.3. Testing of Proposed Method

#### 5.3.1. Comparison of Weighted SUM and Weighted PRODUCT Method

In our research, the accuracy of gaze estimation was measured based on strictly correct estimation rate (SCER) and loosely correct estimation rate (LCER). SCER refers to the ratio of the number of strictly correct frames divided by the number of total frames. The strictly correct frame indicates the frame where the estimated gaze zone is equivalent to the ground truth gaze zone. LCER refers to the ratio of the number of loosely correct frames divided by the number of total frames. The loosely correct frame indicates the frame where the estimated gaze zone is placed within ground truth gaze zone or its surrounding zones. For example, when a driver looked at zone 10 of Figure 3a in a test data image, the SCER considered it correct estimation only when the minimum distance fell to position 10. On the other hand, the LCER considered it a correct estimation when the minimum distance fell to either position 10 or one of the positions in its vicinity—6 through 14.

As explained in Section 4.4.4, Euclidean distances (scores) of inputs are combined by weight SUM or weight PRODUCT rules. The optimal weights for these rules were experimentally determined with training data. It is observed that optimal weights obtained for face, left eye, and right eye using weighted SUM rule in case of scheme 1 are 0.1, 0.5 and 0.4, respectively, with average SCER and LCER value of 92.8% and 99.6% respectively. The optimal weights obtained for face, left eye, right eye, PCCR vector of left eye, and PCCR vector of right eye using weighted SUM rule in case of scheme 2 are 0.085, 0.495, 0.4, 0.011, and 0.009 respectively, with average SCER and LCER value of 64.8% and 91.1% respectively. It is found that optimal weights obtained for face, left eye, and right eye using weighted PRODUCT method in case of scheme 1 are 0.1, 0.5 and 0.4, respectively with average SCER and LCER value of 90.8% and 99.1% respectively. In case of scheme 2, the average SCER and LCER value of 65.7% and 90.4% with optimal weights of 0.09, 0.49, 0.4, 0.01, and 0.01 for face, left eye, right eye, PCCR vector of left eye, and PCCR vector of right eye respectively using weighted PRODUCT rule. Because other parts of the face such as lips can be changed according to the change of facial expression even in case of gazing at the same zone, lower weight was determined for face compared to those for left and right eyes. As shown in Figure 3b, the head rotation in the right direction is more severe than that in the left direction. Therefore, the left eye can be better observed by our gaze-tracking camera (installed in the vicinity of the dashboard, as shown in Figure 2) than right eye, and more gaze information can be obtained from left eye for the driver gaze classification system. Consequently, larger weight was determined for left eye than right eye. In addition, because the weighted SUM rule outperformed the weighted PRODUCT rule, we use the weighted SUM rule in our research.

#### 5.3.2. Comparison of Scheme 1 and Scheme 2

As the next experiment, we have made the comparison of accuracies of gaze estimation by schemes 1 and 2 of Figure 7 and Figure 8. We have analyzed the testing results through different metrics such as the confusion matrix and estimation rate of SCER and LCER. First, we discuss the results obtained through the confusion matrix. It is a popular metric for classification problems on a set of test data for which true values are known. We analyzed the results without using PCCR vectors i.e., scheme 1 explained in Figure 7. As we have collected the results from two-fold cross validation, Table 5 shows the average classification accuracy results from two-fold cross validations (scheme 1). “Actual” and “Predicted” mean the ground-truth and estimated gaze zone, respectively. Observe from the results through the confusion matrix of scheme 1 that almost all the gaze regions have shown a high level of accuracy. Although the distances between gaze regions were small and the number of gaze regions is large, our proposed method has demonstrated high accuracy of gaze estimation. Later, we obtained the results by using scheme 2 for the driver’s gaze classification. We have obtained the results with PCCR vectors from left and right eye combined with face, left, and right eye images. For comparison purposes, we have extracted the results and represented them in the form of the confusion matrix shown below. As we have collected the results from two-fold cross validation, Table 6 shows the average classification accuracy from two-fold cross validation with PCCR vector (scheme 2). 

Observe from the results through the confusion matrix of scheme 2 that the accuracy for driver’s gaze classification in the car environment was degraded with considering PCCR vector (scheme 2) in the results compared to scheme 1. It shows that error in detection of the pupil center and corneal reflection causes the error in PCCR vector, which decreases the accuracy of gaze estimation.

To further verify our results for scheme 1 and scheme 2, we used other metrics for classification i.e., estimation rate categorized into SCER and LCER. Estimation rate is measured using the proposed method without PCCR vectors (scheme 1) and with PCCR (scheme 2) are shown in Table 7 and Table 8 respectively. Table 7 shows the SCER and the LCER results for each gaze region without using PCCR vector (scheme 1).

Note that the average detection rate using SCER is 92.8% and LCER is 99.6%. Even in the case that one of the driver’s eyes is occluded by severe head rotation (gaze regions of 1, 5, and 16 of Figure 3a), our system shows high accuracy of gaze estimation because our system uses the information of the whole face, as shown in Figure 7. Table 8 shows the SCER and the LCER results using PCCR vector (scheme 2).

Note that the average detection rate with PCCR vector (scheme 2) using SCER is 64.8% and LCER is 91.1%. These accuracies are lower than those without PCCR vector (scheme 1). Therefore, we found that it is difficult to use PCCR vector in the outdoor environment using one camera and without driver calibration. It also shows that error in detection of the pupil center and corneal reflection causes the error in PCCR vector, which reduces the accuracy of gaze estimation.

**Figure 13:**
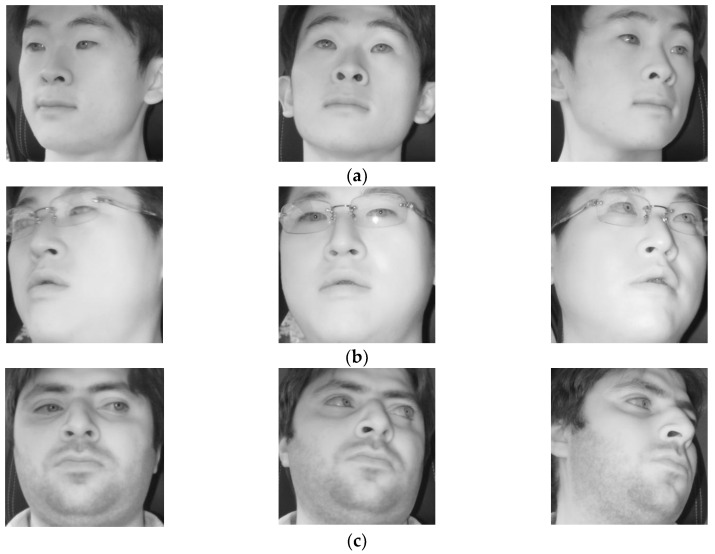
Correctly detected gaze zones with our system. Left, middle, and right figures respectively show the cases that the driver looks at gaze zones of (**a**) 1, 6, and 14; (**b**) 15, 2, and 7; (**c**) 3, 8, and 16.

In Figure 13, we have shown some examples of the correctly classified gaze zones in terms of SCER by our proposed method (scheme 1). As shown in this figure, although the images are collected from different people with different head and eyes directions, our proposed method can classify gaze zone with a high level of accuracy.

**Figure 14:**
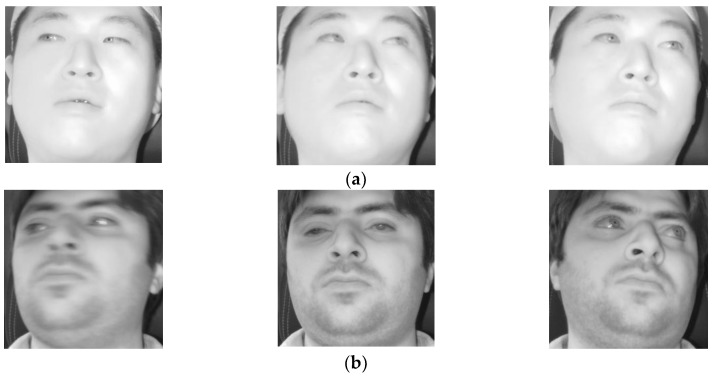

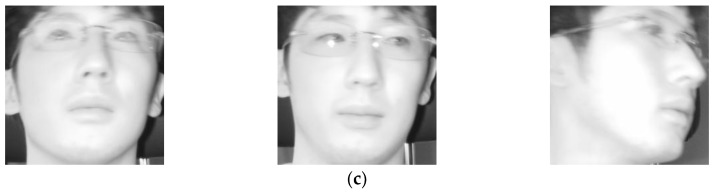
Incorrectly detected gaze zones with our system. Left, middle, and right figures respectively show the cases that the driver looks at gaze zones of (**a**) 1, 7, and 14; (**b**) 15, 2, and 8; (**c**) 9, 3, and 16.

In Figure 14, we have shown some examples of the incorrectly classified gaze zones in terms of SCER by our proposed method (scheme 1). These errors are caused by the variation of head and eye rotations even with gazing at the same zone (for example, by comparing the left figure of Figure 13a and Figure 14a for zone 1, or the right figure of Figure 13c and Figure 14c for zone 16), image blurring, and the eye blinking.

#### 5.3.3. Comparison with Previous Method

In the next experiment, we compared the performance of our proposed method with a previous method [38], where AlexNet CNN model [69] was used to detect eight gaze zones. AlexNet is comprised of five convolutional layers and three fully connected layers. In the first convolutional layer AlexNet uses 96 filters of size 11 × 11 × 3, and uses a local response normalization (LRN) layer after a ReLU layer. Based on Gaussian distribution, the weights in each layer were initialized to random values with standard deviations of 0.01 with a mean of zero [69]. We have detected 17 gaze zones using the previous method. In the previous method [38], they have only considered the face as an input. However, in our method (scheme 1), we used three inputs i.e., face, left eye, and right eye images. We have used the same training and testing data from two-fold cross validation for fair comparison. As we did for comparison between scheme 1 and scheme 2, we have adopted the same metrics, confusion matrix and estimation rate of SCER and LCER. Table 9 shows the average confusion matrix with testing data by the previous method [38] from two-fold cross validation.

Observe from the obtained results that the accuracies by previous method [38] are lower than those by our method when it is tested on DDGC-DB1, covering all 17 gaze regions. As shown in Table 9, the highest accuracy achieved by the previous method based on AlexNet is 72.6% at gaze region 14. That is lower than the accuracy by our method, which achieved an accuracy above 90% in most gaze regions as shown in Table 7. In Table 10, we compared the accuracies by our method and previous method [38] in terms of SCER and LCER.

Note that with previous method [38], the average SCER and LCER is 64.3% and 87.2% respectively. These are lower than the average SCER and LCER obtained from the proposed method: 92.8% and 99.6% respectively. Hence, we can find that the proposed driver gaze classification of scheme 1 of Figure 7 had higher performance and accuracy compared to the previous method based on AlexNet.

#### 5.3.4. Comparison with Open Database

**Figure 15:**
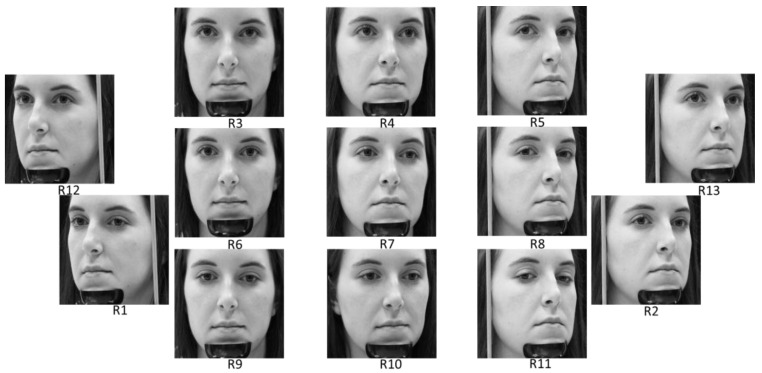
Eye gaze and head pose images selected from CAVE-DB.

In the next experiment, we compared the accuracies by our method with those by previous method [38] on open Columbia gaze dataset CAVE-DB [77]. It is a large gaze database of 56 people with 5880 images over varying head poses and gaze directions. There are 105 gaze directions as 5 head poses with 21 gaze directions per head pose. By excluding the images of severe gaze direction, for all people, we have chosen 13 gaze direction images considering the driver’s gaze in the car environment of Figure 3. The examples of images with gaze zones are shown in Figure 15.

We have obtained augmented data from the selected data for making a fair comparison. Then we performed two-fold cross validation similar to the experiments with DDGC-DB1. Augmented data was used only for training and original data was used for testing similar to the experiments with DDGC-DB1. Results are also summarized in the form of a confusion matrix and estimation rates of SCER and LCER. First, we show results obtained by previous method [38]. Table 11 shows the average confusion matrix obtained from the first- and second-fold cross validation by the previous method using open database where Table 12 shows the same for the proposed method.

It can be analyzed from the obtained results that our proposed method has shown better accuracy on CAVE-DB as compared to the previous method [38]. As can be seen in Table 12, the highest accuracy achieved by our method is 88.9% at gaze region 12. That is much higher than that of previous method i.e., 70.1% at gaze region 12 as shown in Table 11. We further compared the accuracies by previous method [38] and the proposed method through average estimation rate SCER and LCER from two-fold cross validation as shown in Table 13.

Note that with CAVE-DB, average SCER and LCER by the proposed method are 77.7% and 96.3% respectively. This is higher than those of the previous method i.e., 53.1% and 88.7%, respectively. Hence, we find that the proposed driver gaze classification of scheme 1 of Figure 7 had higher performance and accuracy compared to previous method [38] based on AlexNet on CAVE-DB.

**Figure 16:**
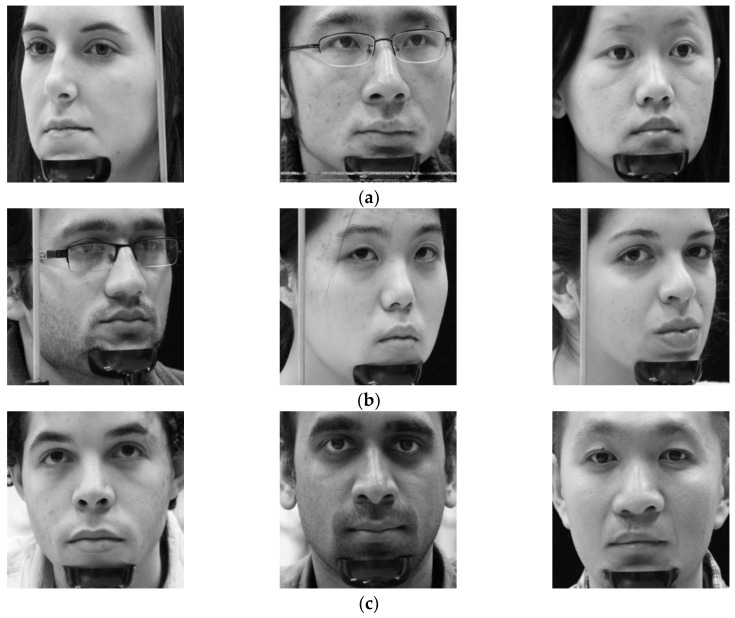
Correctly detected gaze zones with our system on CAVE-DB. Left, middle, and right figures respectively show the cases that the user looks at gaze zones of (**a**) 1, 4, and 7; (**b**) 2, 5, and 8; (**c**) 3, 6, and 9, respectively, of Figure 15.

In Figure 16, we have shown some examples of the correctly classified gaze zones in terms of SCER by our proposed method (scheme 1). As shown in this figure, although the images are collected from different people with different head and eyes directions, our proposed method can classify gaze zone with a high level of accuracy.

**Figure 17:**
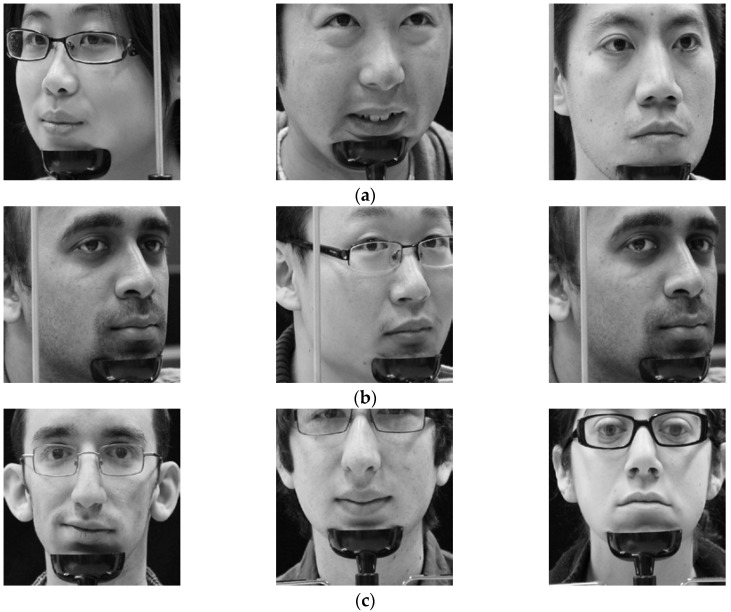
Incorrectly detected gaze zones with our system on CAVE-DB. Left, middle, and right figures respectively show the cases that user looks at gaze zones of (**a**) 1, 4, and 7; (**b**) 2, 5, and 8; (**c**) 3, 6, and 9, respectively, of Figure 15.

In Figure 17, we have shown some examples of the incorrectly classified gaze zones in terms of SCER by our proposed method (scheme 1). Observe that these errors are caused by the variation of head and eye rotations even with gazing at the same zone (for example, by comparing the left figure of Figure 16b and Figure 17b for zone 2). Another reason of error cases is incorrect detection of facial landmarks due to face fixture used in CAVE-DB as shown in the center figure of Figure 17c. 

As the last experiment, we measured the processing speed by our method on DDGC-DB1 and CAVE-DB. Experimental results showed that the average processing time on DDGC-DB1 was 12.72 msec. per image and that on CAVE-DB was 11.21 msec. per image. From that, we can find that our system can be operated at a speed of 78.6 (1000/12.72)~89.2 (1000/11.21) frames per second.

#### 5.3.5. Effect of the Errors in Facial Landmark Detection on the Accuracies of Gaze Detection

We checked how resistant our method is to the potential errors in facial landmark detection. In our method, the regions of two eyes and face for CNN input of Figure 7 are determined by the positions of facial landmarks as shown in Figure 1 (step (3)). Therefore, the errors in facial landmark detection cause the errors of regions of two eyes and face for CNN input of Figure 7. We measured the accuracies by our gaze detection method according to the errors in facial landmark positions on DDGC-DB1 database. As shown in Table 14, in case the errors are less than ±8 pixels in X- and Y-axes, the accuracy degradation by our method is very small (degradation of 0.9% in SCER and that of 0.5% in LCER compared to the accuracies in case of no detection error in facial landmarks). However, in case of the errors same to (or larger than) ±8 pixels, the accuracy degradation is increased (degradation larger than about 3% in SCER and that larger than about 2.1% in LCER from ±8 to ±10 pixels). From that, we can find that our method is resistant to the errors (same to (or less than) ±7 pixels in X- and Y-axes) in facial landmark detection.

#### 5.3.6. Eye Safety

We measured the level of danger by our NIR illuminator. For that, we measured the eye safety of the NIR illuminator based on the American Council of Government and Industrial Hygienists (ACGIH) and threshold limit values (TLV) [78,79]. The ACGIH exposure limit for infrared radiation is defined by the following equation. For exposures greater than 1000 s, irradiance must be limited to less than 10 mW/cm^2^ [78,79]:

(10) $$\overset{3000\;\mathrm{nm}}{\underset{700\;\mathrm{nm}}{\sum }}E_{\lambda }\cdot \Delta \lambda ⩽1.8t^{-\frac{3}{4}}W/{\mathrm{cm}}^{2}$$

where *λ* represents the wavelength of incident light, summation is over the *λ* range where the light level is significant, *E_λ_* represents the irradiance into the eye in W/cm^2^, and *t* represents the exposure time in second. In the proposed system, the exposure time *t* by NIR illuminator was a maximum of 900 s (time-out), and the NIR illuminator is automatically turned off for 0.1 s. Then, it is turned on for 900 s again, and this procedure of turning on and off is iterated. Thus, we obtained the maximum ACGIH exposure limits for infrared radiation as about 10.95 (=1.8 × 900^−3/4^) mW/cm^2^ based on Equation (10). The experimental results showed that the infrared radiation power (0.53 mW/cm^2^) of our NIR illuminator was much less than the limit, so the proposed system met the safety requirements.

## 6. Conclusions

In this study, we proposed a method of driver gaze classification in the vehicular environment based on CNN. For driver gaze classification, face, left eye, and right eye images are obtained from input image based on the ROI defined by facial landmarks from the Dlib facial feature tracker. We performed fine tuning with a pre-trained CNN model separately for the extracted cropped images of face, left eye, and right eye using VGG-face network to obtain the required gaze features from the fully connected layer of the network. Three distances based on all the obtained features are combined to find the final result of classification. The impact of PCCR vector on gaze classification is also studied. We compared the performance of the proposed gaze classification method using CNN with PCCR vector and without PCCR vector. We verified from the results that the driver gaze classification without PCCR vector is suitable in terms of accuracy. We also compared the accuracies of our method with those of a previous method. Evaluations were also performed on open CAVE-DB, and we can confirm that our method outperformed the previous method. Based on the processing time, we can find that our system can be operated at a speed of 78.6~89.2 frames per second.

As shown in Figure 4, the Dlib facial feature tracker cannot detect the position of the pupil and iris. Therefore, in case the driver gazes at a position just by eye movement (not by head movement) when gazing at the position close to our gaze-tracking camera, the method using only facial landmarks by the Dlib facial feature tracker cannot detect accurate gaze position. To solve this problem, the pupil center and corneal reflection position are detected by the method outlined in Section 4.3, and PCCR vector was used for scheme 2 of Figure 8. However, the accuracy of scheme 2 is lower than that of scheme 1 not using PCCR vector as shown in Table 7 and Table 8.

The reason why we used a NIR camera and illuminator is to use the movement of the pupil within eye region (iris region) for gaze estimation for better accuracy. However, our method can also be applied to the images by visible light camera without an additional illuminator, which was proved by the experiments with open Columbia gaze dataset CAVE-DB [77] as explained in Section 5.3.4. In case of severe head and eye rotation, which causes disappearance of one of two eyes in the captured image, the error of gaze estimation can be increased, and this is the limitation of our research. This can be solved by using multiple cameras, but it can also increase the processing time. We would research a solution to this problem by using multiple cameras at fast processing speeds in future work. In addition, we would check the effect of image resolution, blurring level, or severe occlusion on the face image on the accuracy of the gaze estimator.

## Figures and Tables

**Table 1:** Comparison between the proposed and previous research on gaze classification in vehicle environment.

Category	Methods	Advantage	Disadvantage
Multiple camera-based [ 21,22,23,24]	Multiple cameras are used to classify driver’s gaze region	- Possibility of invisible eye region is reduced	- The processing time is increased by the images of multiple cameras
		- Reliability is higher when information from multiple cameras is combined	- Difficulties in applying to actual vehicular environment due to complicated, time-consuming calibration [ 21,22]
Single camera-based	Using SVM [ 27], SDM with SIFT [28], skin color [29,30], HOG, SVM, random forest classifier [31], Purkinje image [35], facial feature points [36], and particle filtering [40]	Computational complexity is lower than multiple camera-based system	Higher possibility of invisible eye regions or disappearance of pupil and CR, which can negatively affect the reliability of system
	Regression-based method [ 41,42]	Continuous gaze position can be obtained instead of discrete one	
	Using one CNN based on driver’s face image [ 38,39]	- Accurate detection of pupil and CR is not required	- Small numbers of gaze regions are covered
		- Driver’s calibration is not required for gaze classification	- Large quantity of data and time is required to train CNN
	Using deep three CNNs based on driver’s eyes and face images (Proposed method)	- Accurate detection of pupil and CR is not required	Large quantity of data and time is required to train CNN
		- Driver’s calibration is not required for gaze classification	
		- Large numbers of gaze regions are covered	

**Table 2:** Specification of illuminator and camera in our system.

**NIR LED Illuminator**	
Wavelength	850 nm
Number of NIR LEDs	6
**USB Camera**	
Product name	ELP-USB500W02M-L36 [55]
Spatial resolution of image	1600 × 1200 pixels

**Table 3:** Configuration of CNN model used in our research (CL means convolutional layer).

Layer Types		Number of Filters	Size of Feature Map	Size of Kernel	Stride Number	Padding Number
Image input layer		-	224 (height) × 224 (width) × 3 (channel)	-	-	-
Group 1	Conv-1_1 (1st CL)	64	224 × 224 × 64	3 × 3	1 × 1	1 × 1
	ReLU-1_1	-	224 × 224 × 64	-	-	-
	Conv-1_2 (2nd CL)	64	224 × 224 × 64	3 × 3	1 × 1	1 × 1
	ReLU-1_2	-	224 × 224 × 64	-	-	-
	Pool-1	1	112 × 112 × 64	2 × 2	2 × 2	0 × 0
Group 2	Conv-2_1 (3rd CL)	128	112 × 112 × 128	3 × 3	1 × 1	1 × 1
	ReLU-2_1	-	112 × 112 × 128	-	-	-
	Conv-2_2 (4th CL)	128	112 × 112 × 128	3 × 3	1 × 1	1 × 1
	ReLU-2_2	-	112 × 112 × 128	-	-	-
	Pool-2	1	56 × 56 × 128	2 × 2	2 × 2	0 × 0
Group 3	Conv-3_1 (5th CL)	256	56 × 56 × 256	3 × 3	1 × 1	1 × 1
	ReLU-3_1	-	56 × 56 × 256	-	-	-
	Conv-3_2 (6th CL)	256	56 × 56 × 256	3 × 3	1 × 1	1 × 1
	ReLU-3_2	-	56 × 56 × 256	-	-	-
	Conv-3_3 (7th CL)	256	56 × 56 × 256	3 × 3	1 × 1	1 × 1
	ReLU-3_3	-	56 × 56 × 256	-	-	-
	Pool-3	1	28 × 28 × 246	2 × 2	2 × 2	0 × 0
Group 4	Conv-4_1 (8th CL)	512	28 × 28 × 512	3 × 3	1 × 1	1 × 1
	ReLU-4_1	-	28 × 28 × 512	-	-	-
	Conv-4_2 (9th CL)	512	28 × 28 × 512	3 × 3	1 × 1	1 × 1
	ReLU-4_2	-	28 × 28 × 512	-	-	-
	Conv-4_3 (10th CL)	512	28 × 28 × 512	3 × 3	1 × 1	1 × 1
	ReLU-4_3	-	28 × 28 × 512	-	-	-
	Pool-4	1	14 × 14 × 512	2 × 2	2 × 2	0 × 0
Group 5	Conv-5_1 (11th CL)	512	14 × 14 × 512	3 × 3	1 × 1	1 × 1
	ReLU-5_1	-	14 × 14 × 512	-	-	-
	Conv-5_2 (12th CL)	512	14 × 14 × 512	3 × 3	1 × 1	1 × 1
	ReLU-5_2	-	14 × 14 × 512	-	-	-
	Conv-5_3 (13th CL)	512	14 × 14 × 512	3 × 3	1 × 1	1 × 1
	ReLU-5_3	-	14 × 14 × 512	-	-	-
	Pool-5	1	7 × 7 × 512	2 × 2	2 × 2	0 × 0
Fc6 (1st FCL)		-	4096 × 1	-	-	-
ReLU-6		-	4096 × 1	-	-	-
Dropout-6		-	4096 × 1	-	-	-
Fc7 (2nd FCL)		-	4096 × 1	-	-	-
ReLU-7		-	4096 × 1	-	-	-
Dropout-7		-	4096 × 1	-	-	-
Fc8 (3rd FCL)		-	17 × 1	-	-	-
Softmax layer		-	17 × 1	-	-	-
Output layer		-	17 × 1	-	-	-

**Table 4:** Description of training and testing images from DDGC-DB1.

Two-Fold Cross Validation	Training	Testing
1st fold cross validation	16,310 (3262 × 5) images for each sub-database (face, left, and right eyes) from 10 people	3256 images for each sub-database (face, left, and right eyes) from 10 people
2nd fold cross validation	16,280 (3256 × 5) images for each sub-database (face, left, and right eyes) from 10 people	3262 images for each sub-database (face, left, and right eyes) from 10 people

**Table 5:** Average confusion matrix of scheme 1 from two-fold cross validation.

Predicted																		
**Actual**		**R1**	**R2**	**R3**	**R4**	**R5**	**R6**	**R7**	**R8**	**R9**	**R10**	**R11**	**R12**	**R13**	**R14**	**R15**	**R16**	**R17**
	**R1**	98.8	0.4	-	-	-	-	-	-	-	-	-	0.3	-	-	0.3	-	0.2
	**R2**	1	97.1	0.6	-	-	-	-	-	-	-	-	0.6	0.2	-	0.2	-	0.3
	**R3**	-	0.8	97.4	0.8	-	-	-	-	-	-	-	0.2	0.3	0.1	-	-	0.4
	**R4**	-	-	1.2	96.9	0.9	-	-	-	-	-	-	-	0.4	0.2	-	0.4	-
	**R5**	-	-	-	2.9	91.4	-	-	-	-	-	-	-	-	2.9	-	2.8	-
	**R6**	-	-	-	-	-	95	1.4	0.1	2	0.8	-	0.1	-	-	0.6	-	-
	**R7**	-	-	0.2	-	-	2.1	89.1	2.9	1.4	1.8	1.3	0.6	0.2	-	0.4	-	-
	**R8**	-	0.1	-	0.5	-	-	2.3	87.5	1.6	2	2.3	1.2	-	0.6	-	1.9	-
	**R9**	-	-	-	-	-	1.9	1.6	-	90.4	1.8	-	1.9	1.2	0.1	1.1	-	-
	**R10**	-	-	-	-	-	0.9	1.1	0.5	0.4	94.9	0.9	0.5	0.4	0.4	-	-	-
	**R11**	-	-	-	-	-	-	1	1.8	-	0.8	92.2	0.2	1.3	1.5	-	1.2	-
	**R12**	0.9	1.3	1	-	-	-	-	-	1.7	2.3	-	89.7	1.3	-	1.8	-	-
	**R13**	-	0.3	0.6	0.5	-	-	-	-	0.9	0.7	0.7	0.9	94.7	0.7	-	-	-
	**R14**	-	-	0.8	1.4	2.3	0.1	-	0.1	-	0.9	1.8	-	1.1	89.7	-	1.8	-
	**R15**	4.4	-	-	-	-	4.4	-	-	4.4	-	-	5.5	-	-	81.3	-	-
	**R16**	-	-	-	-	1.5	-	-	1.4	-	-	1.9	-	-	1.2	-	94	-
	**R17**	0.8	1.2	1.1	-	-	-	-	-	-	-	-	-	-	-	-	-	96.9

**Table 6:** Average confusion matrix of scheme 2 from two-fold cross validation.

	Predicted																	
**Actual**		**R1**	**R2**	**R3**	**R4**	**R5**	**R6**	**R7**	**R8**	**R9**	**R10**	**R11**	**R12**	**R13**	**R14**	**R15**	**R16**	**R17**
	**R1**	58.6	6.1	4.2	-	-	3.9	-	-	1.1	-	-	8.7	-	-	8.1	-	9.3
	**R2**	3.9	66.8	3.2	-	3.8	-	1.8	2	2.3	0.3	-	6	4.5	-	2.7	-	2.7
	**R3**	1.2	3	66	3.1	-	1.1	1.6	1.6	1.8	-	2.6	5.7	3.6	3	-	-	5.7
	**R4**	0.9	-	6.9	60.7	6.3	-	1.2	-	0.5	-	0.6	0.5	6.8	7.4	0.7	7.5	-
	**R5**	-	0.8	0.6	11.8	61.4	0.1	-	1.5	-	3.3	-	0.9	0.2	10.2	-	9.2	-
	**R6**	1	-	2.3	-	0.8	73.7	1.6	0.6	5.3	5.5	0.2	0.9	2.3	-	5.8	-	-
	**R7**	1.6	-	2.6	0.1	-	4.6	68.2	4.5	2.5	3.8	3.1	1.2	4.1	-	1.4	2.3	-
	**R8**	-	2	-	1.6	-	0.5	3.2	73	0.2	3.8	3.4	-	-	5.2	-	6.2	0.9
	**R9**	-	1	3	2.3	0.7	1	4	0.6	67.7	3.9	2	5.4	5.1	0.4	2.2	0.7	-
	**R10**	1.5	-	2.9	1.6	3.9	4.8	2.7	1.5	3.6	61.3	3.6	3.9	4.4	3.6	0.2	-	0.5
	**R11**	1.6	2	1.1	0.1	0.6	-	6.7	5.1	0.7	2.4	61.8	-	6.2	4.3	0.8	5.3	1.3
	**R12**	1.4	2.7	3.1	-	2.4	-	2.8	1.7	5.6	4.5	0.2	67.4	3.2	0.6	3.4	-	1
	**R13**	-	3.3	3.5	2.7	0.7	1.2	0.2	1.4	2.2	2.1	2.7	3.3	68	4.1	1.3	1.4	1.9
	**R14**	-	-	3.2	5.3	4.8	-	1.3	2	0.3	3.8	5.5	-	6	59.4	-	6.9	1.5
	**R15**	8.4	1.5	0.2	-	-	6.8	-	1.6	8.1	-	-	6.9	-	-	66	-	0.5
	**R16**	0.4	1.8	0.6	0.2	6.9	-	2.5	7.9	0.8	-	7.1	1.5	0.1	9	-	61.2	-
	**R17**	9	9	6.9	0.8	2.5	6.3	-	-	0.8	-	0.9	0.8	0.5	2.5	-	-	60

**Table 7:** SCER and LCER of scheme 1 without PCCR vectors.

Gaze Regions	Neighbors	SCER (%)	LCER (%)
1	2,12,15,17	98.8	100
2	1,3,12,13,15,17	97.1	100
3	2,4,12,13,14,17	97.4	100
4	3,5,13,14,16	96.9	100
5	4,14,16	91.4	100
6	7,9,10,15	95	99.8
7	6,8,9,10,11	89.1	98.6
8	7,10,11,16	87.5	96
9	6,7,10,12,13,15	90.4	99.9
10	6,7,8,9,11,12,13,14	94.9	100
11	7,8,10,13,14,16	92.2	99.8
12	1,2,3,9,10,13,15	89.7	100
13	2,3,4,9,10,11,12,14	94.7	100
14	3,4,5,10,11,13,16	89.7	99.8
15	1,6,9,12	81.3	100
16	5,8,11,14	94	100
17	1,2,3	96.9	100
Average		**92.8**	**99.6**

**Table 8:** SCER and LCER of scheme 2 with PCCR vectors.

Target Zone	Neighbors	SCER (%)	LCER (%)
1	2,12,15,17	58.6	90.8
2	1,3,12,13,15,17	66.8	89.8
3	2,4,12,13,14,17	66	90.1
4	3,5,13,14,16	60.7	95.6
5	4,14,16	61.4	92.6
6	7,9,10,15	73.7	91.9
7	6,8,9,10,11	68.2	86.7
8	7,10,11,16	73	89.6
9	6,7,10,12,13,15	67.7	89.3
10	6,7,8,9,11,12,13,14	61.3	89.4
11	7,8,10,13,14,16	61.8	91.8
12	1,2,3,9,10,13,15	67.4	91.3
13	2,3,4,9,10,11,12,14	68	91.9
14	3,4,5,10,11,13, 16	59.4	94.9
15	1,6,9,12	66	96.2
16	5,8,11,14	61.2	92.1
17	1,2,3	60	84.9
Average		**64.8**	**91.1**

**Table 9:** Average confusion matrix of previous method [38] from two-fold cross validation.

	Predicted																	
**Actual**		**R1**	**R2**	**R3**	**R4**	**R5**	**R6**	**R7**	**R8**	**R9**	**R10**	**R11**	**R12**	**R13**	**R14**	**R15**	**R16**	**R17**
	**R1**	68.4	2	-	-	-	4	1.9	0.2	-	-	-	8.2	-	-	11.8	-	3.5
	**R2**	1	65.3	5	-	-	-	-	0.3	-	-	-	16.3	-	-	1.5	1.7	8.9
	**R3**	-	2.6	57.6	7.4	6.8	-	-	0.9	-	-	-	11.5	4.5	0.5	-	7.5	0.7
	**R4**	-	-	9.8	68.3	11.3	1.8	0.5	-	-	0.8	-	-	0.5	-	-	7	-
	**R5**	-	-	-	1.3	68.1	6.7	-	4	-	-	1.4	0.3	-	6.2	-	12	-
	**R6**	-	-	0.5	0.5	1.5	55.2	11.6	-	15.5	9.2	-	4	1.7	-	-	0.3	-
	**R7**	-	-	-	9.6	0.1	0.8	62.3	5.6	-	11	-	-	9.8	0.8	-	-	-
	**R8**		-	-	0.8	8.5	5.3	7.2	62.3	2.5	-	0.6	-	0.8	4	-	8	-
	**R9**	-	6.5	-	-	1	5	1.9	0.3	64.4	5.9	-	6.8	2.6	-	0.3	-	5.3
	**R10**	-	-	0.1	-	0.7	8.8	4.9	-	0.5	51.2	-	0.3	12.1	12.1	-	8.2	1.1
	**R11**	-	-	-	5.7	-	-	-	10.9	-	10.2	68.3	-	4.3	0.3	-	0.3	-
	**R12**	-	6.8	0.3	0.3	1	6.5	2	-	4.9	-	-	60.5	1.4	-	3.8	4.9	7.6
	**R13**	-	-	1.4	3.5	-	10	7.1	-	1.1	2.1	-	0.3	68.2	4.4	0.3	1.1	0.5
	**R14**	-	-	4.3	9.6	3.3	4.5	0.5	-	-	1.5	1.5	-	2.2	72.6	-	-	-
	**R15**	6	3.9	7.6	4.5	-	0.5	-	-	0.3	4.1	-	0.5	0.3	-	67.4	1.6	3.3
	**R16**	-	0.9	0.5	2.5	16.9	0.9	-	3.9	0.6	0.3	1.1	-	0.7	-	0.7	70.7	0.3
	**R17**	11.4	6.9	0.3	-	0.3	1.5	5.1	-	0.3	1.4	-	-	0.5	-	5.7	4.9	61.7

**Table 10:** SCER and LCER of previous method [38] and proposed method on DDGC-DB1.

Gaze Regions	Neighbors	Previous Method [38]		Proposed Method	
		SCER (%)	LCER (%)	SCER (%)	LCER (%)
1	2,12,15,17	68.4	93.9	98.8	100
2	1,3,12,13,15,17	65.3	98	97.1	100
3	2,4,12,13,14,17	57.6	84.8	97.4	100
4	3,5,13,14,16	68.3	96.9	96.9	100
5	4,14,16	68.1	87.6	91.4	100
6	7,9,10,15	55.2	91.5	95	99.8
7	6,8,9,10,11	62.3	79.7	89.1	98.6
8	7,10,11,16	62.3	78.1	87.5	96
9	6,7,10,12,13,15	64.4	86.9	90.4	99.9
10	6,7,8,9,11,12,13,14	51.2	89.9	94.9	100
11	7,8,10,13,14,16	68.3	94.3	92.2	99.8
12	1,2,3,9,10,13,15	60.5	77.7	89.7	100
13	2,3,4,9,10,11,12,14	68.2	81	94.7	100
14	3,4,5,10,11,13,16	72.6	95	89.7	99.8
15	1,6,9,12	67.4	74.7	81.3	100
16	5,8,11,14	70.7	92.6	94	100
17	1,2,3	61.7	80.3	96.9	100
Average		**64.3**	**87.2**	**92.8**	**99.6**

**Table 11:** Average confusion matrix of previous method [38] on CAVE-DB from two-fold cross validation.

	Predicted													
**Actual**		**R1**	**R2**	**R3**	**R4**	**R5**	**R6**	**R7**	**R8**	**R9**	**R10**	**R11**	**R12**	**R13**
	**R1**	53.5	-	1.8	-	1.8	-	-	-	1.8	-	-	41.1	-
	**R2**	-	53.3	-	5.4	20.4	-	-	2.9	-	1.8	7.2	-	9
	**R3**	-	-	51.8	1.8	-	21.4	1.8	-	23.2	-	-	-	-
	**R4**	-	-	-	51.7	7.1	-	23.3	1.8	1.8	12.5	1.8	-	-
	**R5**	-	4	-	5.4	53	-	-	16	1.8	-	10.8	-	9
	**R6**	-	-	21.8	-	-	52.1	1.8	-	20.7	1.8	-	-	1.8
	**R7**	-	-	-	18.2	2.9	-	47.7	1.8	3.6	24	1.8	-	-
	**R8**	-	4	1.8	5.4	16.9	-	-	45.5	-	1.8	21	-	3.6
	**R9**	-	-	14.3	-	-	19.7	-	-	55.5	8.7	-	-	1.8
	**R10**	1.8	-	-	10.7	1.8	1.8	21.4	3.6	3.6	53.5	1.8	-	-
	**R11**	-	7.1	-	1.8	14.3	-	-	13.4	-	5.4	52.6	-	5.4
	**R12**	21.5	-	-	-	4.8	-	-	1.8	1.8	-	-	70.1	-
	**R13**	-	16.1	-	3.6	9	-	-	12.5	-	-	8.9	-	49.9

**Table 12:** Average confusion matrix of proposed method on CAVE-DB from two-fold cross validation.

	Predicted													
**Actual**		**R1**	**R2**	**R3**	**R4**	**R5**	**R6**	**R7**	**R8**	**R9**	**R10**	**R11**	**R12**	**R13**
	**R1**	86.2	-	1.8	-	-	2.8	0.2	-	3.8	0.7	-	4.5	-
	**R2**	-	81.9	1.2	0.6	4.2	0.1	0.4	4.9	0.1	0.5	5.1	-	1
	**R3**	1.6	0.5	80.8	5.5	0.9	4.8	2.8	0.1	1.1	0.4	-	0.9	0.6
	**R4**	-	2.7	4.8	72.8	3	2.4	8	4.2	0.4	0.4	0.4	-	0.9
	**R5**	-	3.4	0.5	2	79.1	0.4	1.2	4.3	-	0.2	2.9	-	6
	**R6**	0.7	0.4	3.7	1.7	0.2	73.1	6.4	0.3	7.9	3.2	0.7	0.9	0.8
	**R7**	0.2	1.4	1.7	2.7	1.5	6	67.2	4.1	4.9	6	3.3	0.1	0.9
	**R8**	-	6.9	0.9	2.9	5.1	0.3	2.6	67.2	0.1	3.5	6.3	-	4.2
	**R9**	1.1	-	1.4	0.4	-	3.3	1.3	-	87	4.9	0.6	-	-
	**R10**	-	0.7	0.5	0.9	0.4	3.4	2.8	0.6	7.4	79	4.1	-	0.2
	**R11**	-	8.1	-	1.8	0.3	0.6	6.2	6.8	0.6	5.9	69.4	-	0.3
	**R12**	6.1	-	2.4	-	-	0.9	0.3	0.6	0.5	-	0.2	88.9	0.1
	**R13**	-	7.3	0.6	1.9	4.6	0.3	1.3	3.5	0.3	0.4	2.3	-	77.5

**Table 13:** SCER and LCER of previous [38] and proposed methods on CAVE-DB.

Gaze Regions	Neighbors	Previous Method [38]		Proposed Method	
		SCER (%)	LCER (%)	SCER (%)	LCER (%)
1	6,9,12	53.5	96.4	86.2	97.3
2	8,11,13	53.3	72.4	81.9	92.9
3	4,6,7,12	51.8	76.8	80.8	94.8
4	3,5,6,7,8	51.7	83.9	72.8	95.2
5	4,7,8,13	53	83.4	79.1	92.6
6	1,3,4,7,9,10,12	52.1	98.2	73.1	97.6
7	3,4,5,6,8,9,10,11	47.7	100	67.2	97.4
8	2,4,5,7,10,11,13	45.5	98.2	67.2	98.7
9	1,6,7,10,12	55.5	83.9	87	97.6
10	6,7,8,9,11	53.5	85.7	79	97.3
11	2,7,8,10,13	52.6	83.9	69.4	96.7
12	1,3,6,9	70.1	93.4	88.9	98.8
13	2,5,8,11	49.9	96.4	77.5	95.2
Average		**53.1**	**88.7**	**77.7**	**96.3**

**Table 14:** SCER and LCER of our method according to the errors in facial landmark detection by Dlib facial feature tracker (X-axis of 0 and Y-axis of 0 mean no detection error in facial landmarks).

Detection Error in Facial Landmarks		SCER (%)	LCER (%)
X-Axis (pixels)	Y-Axis (pixels)		
0	0	92.8	99.6
±1	±1	92.8	99.6
±2	±2	92.7	99.5
±3	±3	92.7	99.4
±4	±4	92.5	99.3
±5	±5	92.3	99.3
±6	±6	92.1	99.1
±7	±7	91.9	99.1
±8	±8	88.4	96.7
±9	±9	85.4	94.6
±10	±10	82.2	92.4
